# Bioinspired Deep Neural Networks for Predicting Income-Reporting Discontinuities in the Chilean Student Loan Program

**DOI:** 10.3390/biomimetics11020098

**Published:** 2026-02-01

**Authors:** Yoslandy Lazo, Álex Paz, Broderick Crawford, Carlos Valle, Eduardo Rodriguez-Tello, Ricardo Soto, José Barrera-Garcia, Felipe Cisternas-Caneo, Benjamín López Cortés

**Affiliations:** 1 Escuela de Ingeniería Informática, Pontificia Universidad Católica de Valparaíso, Avenida Brasil 2241, Valparaíso 2362807, Chilericardo.soto@pucv.cl (R.S.); jose.barrera@pucv.cl (J.B.-G.); felipe.cisternas.c@mail.pucv.cl (F.C.-C.); benjamin.lopez.c@mail.pucv.cl (B.L.C.); 2 Escuela de Ingeniería en Construcción y Transporte, Pontificia Universidad Católica de Valparaíso, Avenida Brasil 2147, Valparaíso 2362804, Chile; 3 Cinvestav Unidad Tamaulipas, Km. 5.5 Carretera Victoria-Soto La Marina, Victoria 87130, Tamaulipas, Mexico; ertello@cinvestav.mx; 4 Escuela de Negocios y Economía, Pontificia Universidad Católica de Valparaíso, Amunátegui 1838, Viña del Mar 2580129, Chile

**Keywords:** bioinspired deep neural networks, student credit risk prediction, imbalanced data, model interpretability

## Abstract

This study addresses discontinuity prediction in income reporting within the Chilean student loan program, a critical event for credit risk management. Although the literature has incorporated machine learning models to anticipate non-compliance behavior, a gap remains in the development of methodologically robust evaluations that integrate nonlinear imputation, imbalance correction, and repeated validation across multiple partitions. To address this need, a complete pipeline was implemented on a dataset of 22,303 records, including MissForest imputation, SMOTE-based balancing, and a comparative assessment of a biologically inspired Deep Neural Network (DNN) and a Random Forest (RF) classifier used as a classical baseline model, evaluated across 35 stratified partitions. The results show that the bioinspired DNN, as the primary focus of this study, consistently outperforms the RF in metrics such as AUC (0.9991 vs 0.9709), F1-score (0.9966 vs 0.9497), and agreement measures, while also exhibiting lower variability across partitions. The interpretability analysis indicates that financial variables account for the greatest influence on predictions, whereas demographic variables contribute minimally. The study provides a replicable and robust methodology aligned with risk analysis practices in student credit contexts.

## 1. Introduction

The assessment of credit risk in student financing programs is a fundamental component for ensuring the sustainability of these systems and promoting more efficient management practices [1]. In this context, the early identification of signals associated with critical behaviors, such as discontinuity in income reporting, is essential for anticipating risk areas and strengthening monitoring processes. The literature has indicated that the financial behaviors of beneficiaries may be influenced by demographic, socioeconomic, and administrative factors of a heterogeneous nature, which complicates their modeling through traditional approaches [1,2]. This has fueled interest in developing predictive models capable of capturing complex relationships and providing reliable estimates in scenarios where the available information is diverse and, at times, incomplete [1,2,3,4,5], particularly biologically inspired approaches such as Deep Neural Networks (DNNs), which emulate key aspects of neuronal information processing.

Despite the advances achieved in risk analysis applied to student populations, significant challenges persist that affect the accuracy and practical usefulness of the models. Among these challenges are the presence of missing values in key variables, the mixture of categorical and numerical data, and, especially, the strong class imbalance associated with the behavior to be predicted [5]. These conditions hinder the construction of generalizable models and tend to bias predictions toward the most frequent patterns [1,6]. Likewise, several studies have emphasized the need to develop more comprehensive methodological procedures that not only address these challenges from a preprocessing perspective, but also allow for evaluating model stability under different data partitions and contribute to a better interpretation of the factors influencing predictions [2,3,4]. In the specific case of educational credit programs, these limitations become particularly relevant, given that the adequate identification of beneficiaries with higher potential risk directly impacts the efficiency of the system [1].

Motivated by these challenges, the present study proposes a methodological approach aimed at improving the prediction of discontinuity in income reporting within a student financing program, with a primary focus on a biologically inspired Deep Neural Network (DNN) as the core predictive model. To this end, a complete pipeline is integrated, encompassing rigorous treatment of missing values and the proper preparation of temporal and financial variables, as well as the correction of the marked class imbalance and the systematic evaluation of performance through multiple stratified partitions. In addition, models of different natures are incorporated, including a biologically inspired deep learning architecture (DNN), whose design draws on principles of cerebral information processing, and a tree-based statistical model (RF) used as a classical baseline, in order to analyze whether more expressive architectures are capable of capturing relevant patterns in this type of data. Finally, an interpretability component is included to identify the variables with the greatest influence on the predictions, contributing to a more transparent understanding of the modeled behavior.

Given that the study is grounded in administrative and financial records from the Pontificia Universidad Católica de Valparaíso (PUCV), the proposed approach responds to the specific characteristics of the Chilean student loan model, in which the institutional management of the Fondo Solidario de Crédito Universitario (FSCU) plays a central role in ensuring financial sustainability and adequate monitoring of beneficiaries.

This Chilean case study is particularly relevant for higher education institutions that operate under the FSCU framework, where universities bear the responsibility for credit administration and default mitigation. Therefore, improving predictive capacity in this context has direct implications for institutional financial planning and long-term program sustainability.

The novelty of the proposed approach resides in the integration of a biologically inspired deep neural architecture within a rigorously controlled experimental pipeline. This pipeline is designed to address recurrent methodological limitations in student credit risk modeling, including partition dependence [7], imbalance-induced bias, and limited interpretability [8,9]. Robust multiseed validation, statistical testing, and gradient-based explanations are jointly incorporated to enhance the practical reliability of bioinspired learning systems in educational finance.

The main contributions of this work are:

- The development of a systematic preprocessing pipeline focused on the treatment of missing values, the coherent transformation of variables, and the correction of class imbalance.
- The comparative evaluation of a biologically inspired deep learning model (DNN) and a traditional baseline approach (RF), applied consistently across 35 stratified partitions to ensure robustness in performance estimation.
- The incorporation of interpretability methods that allow identifying the factors with the greatest influence on the predictions, providing a more transparent view of the classification process.
- The presentation of a replicable methodology that integrates good practices in preprocessing, validation, and analysis, contributing to the strengthening of the tools available for predicting student credit risk.

The article is organized as follows. Section 2 presents the review of the relevant literature on credit risk prediction and modeling. Section 3 describes the data used and details the methodological pipeline, including preprocessing, balancing procedures, and the evaluated models. Section 4 reports the results obtained across the different partitions and performance metrics. Section 5 discusses the findings in relation to previous studies and analyzes their implications for risk management in the FSCU. Finally, Section 6 presents the conclusions, the study’s limitations, and potential directions for future research.

## 2. Related Work

The literature on credit risk prediction in student contexts has shown that the financial behavior of beneficiaries exhibits dynamics more complex than those observed in traditional credit systems. Several studies have applied machine learning techniques to anticipate delinquency or default situations, highlighting the use of tree-based models, penalized regressions, and deep neural networks (DNNs), the latter recognized for their biologically inspired capacity to model complex interactions [2,5]. In general terms, these approaches have shown improvements compared to classical statistical methods, particularly in capturing nonlinear relationships and heterogeneous patterns among demographic, academic, and economic variables [5]. However, several studies agree that the effectiveness of these models strongly depends on the quality of preprocessing and the algorithm’s ability to handle incomplete and imbalanced data [10,11,12,13,14].

A relevant body of research has focused on issues of missing values and data heterogeneity, proposing imputation strategies more flexible than mean or mode imputation [15,16]. Among these approaches, methods such as MissForest and other model-based techniques stand out, having demonstrated advantages in scenarios where the relationships among predictor variables are complex and nonlinear [15,17,18]. However, most of these studies are evaluated on single partitions of the dataset, which limits the generalization of their conclusions in the face of partition variability and unstable class distributions [5].

Likewise, recent studies have highlighted the challenge posed by class imbalance in student credit problems, where relevant events are typically rare [5]. Although synthetic oversampling techniques such as SMOTE have been successfully used to mitigate this issue [19,20,21], several authors have warned that their application can introduce risks of overfitting and affect model stability, particularly when combined with highly flexible algorithms such as deep neural networks [19].

On the other hand, biologically inspired deep learning models, due to their capacity to model hierarchical and nonlinear interactions, have been explored in credit scenarios, and some studies have incorporated hyperparameter optimization mechanisms to improve their performance [22,23,24]. However, in many cases, the comparison between classical baseline models and bioinspired deep architectures, such as DNNs, is conducted without adequate control over the variability induced by the partitioning process or without reproducible experimental schemes based on multiple seeds [5,21]. Moreover, interpretability remains a widely noted limitation, especially when models are applied to sensitive financial data such as those linked to student credit programs.

In summary, although the literature has advanced in imputation techniques, handling class imbalance, and the use of complex models for credit risk prediction, there is a clear gap in studies that evaluate these strategies under a robust experimental design that considers multiple partitions, controlled comparisons between biologically inspired models such as DNNs and classical statistical baselines, and interpretability mechanisms that allow validation of prediction consistency. The present work directly addresses this gap through a systematic approach that combines advanced imputation, balancing, hyperparameter optimization, and repeated evaluation over multiple stratified partitions.

## 3. Materials and Methods

### 3.1. Dataset and Data Source

For the development of this research, a dataset provided by the Department of Finance of the Pontificia Universidad Católica de Valparaíso (PUCV) was used, thereby situating the study within a Chilean institutional context directly responsible for administering the FSCU student loan program. This dataset contains historical information related to the Chilean student loan program, *Fondo Solidario de Crédito Universitario* (FSCU). It includes demographic, financial, and credit behavior records of the program’s beneficiary students, structured into seven main tables: Person, Promissory Note, Maturity Group, Debt, Installments, Payment Receipt, and Income Declaration. These data include relevant variables such as gender, date of birth, amount and status of debts, due dates, financial installments, and annual income information, enabling a comprehensive analysis of the debtor’s credit profile. The variables in the dataset are kept in their original language (Spanish) for consistency with the names defined by the source institution. Because the FSCU scheme assigns universities the administrative responsibility for monitoring debtor behavior, the use of institutional data from PUCV provides a realistic setting for analyzing risk-relevant patterns within the Chilean higher-education financing model.

### 3.2. Data Preprocessing

The data preprocessing stage was essential to ensure the quality and suitability of the dataset for training the predictive models. This process encompassed several phases, including cleaning, imputation, transformation, and partitioning of the dataset, as well as handling class imbalance.

#### 3.2.1. Data Cleaning and Missing Value Handling

The cleaning process began with the identification and removal of 107 duplicate records, reducing the total from 22,410 to 22,303 unique records. Subsequently, variables with significant proportions of missing values were identified, such as: sexo (33.24%), fecha_nacimiento (33.39%) and deud_monto_cuota_fija (26.94%). Additionally, the variable deuda_total exhibited 1.51% missing values. These records were removed because, according to the validation team (the Solidarity Fund Unit of PUCV), they correspond to migrations of particular cases within the database. These are atypical cases attributable to administrative processes and are not representative of the regular operation of the system.

Table 1 shows the variables affected by missing values, including their data type, absolute number of missing entries, and percentage relative to the total number of records.

Additionally, five variables with low predictive usefulness were removed, identified according to criteria of extreme imbalance (≥99.5% of a single value), constant values, or a high proportion of zeros (>90%). The eliminated variables were: dein_c_inst_conyuge_deud (95.3% zeros), nacionalidad (99.6% single value), paga_e_pagare (99.9%), deud_t_deuda y paga_t_pagare (both constant). This cleaning step allows reducing dimensionality and avoiding noise in subsequent predictive modeling stages.

#### 3.2.2. Imputation with MissForest

Given the high level of missing values in some key variables, and considering the presence of mixed data types (numerical and categorical), the MissForest algorithm was selected for imputation. This random forest-based technique preserves the natural variability of the data and mitigates biases introduced by simpler methods, such as mean or mode imputation [25]. MissForest is particularly suitable for medium to large datasets and levels of *missingness* above 30%. To avoid data leakage, imputation was performed exclusively using the training set in each partition, after stratified division of the data. Once the imputation model was trained on the training data, the estimated values were applied consistently to the validation and test sets. This strategy ensures that no future information is incorporated into the training process, thus preserving the integrity and validity of the predictive models. MissForest was applied to the variables fecha_nacimiento and deud_monto_cuota_fija, after appropriately transforming them to ensure their compatibility with the algorithm, as described in the variable transformation section. This approach allowed us to leverage MissForest’s potential to recover missing information in key variables without introducing biases derived from inappropriate imputation.

#### 3.2.3. Transformation of Date Variables into Numerical Variables

For the processing of date-type variables, a two-stage transformation was performed. First, prior to imputation, the fecha_nacimiento and deud_fecha_exigibilidad dates were converted to ordinal format (float64), since the MissForest algorithm does not support datetime objects. After imputation, the dates were converted back to the datetime64[ns] format and decomposed into calendar components: year, month, and day. Thus, the variables were nacimiento_anio, nacimiento_mes, nacimiento_dia, and exig_anio. The columns exig_mes and exig_dia were removed as they exhibited no variability (always January), making them irrelevant for modeling.

#### 3.2.4. Logarithmic Transformation of a Skewed Variable

The deuda_total variable exhibited a highly right-skewed distribution, with a skewness coefficient greater than 22. To stabilize its variance, reduce numerical dominance, and improve the model’s ability to capture non-trivial patterns, the adjusted logarithmic transformation log1p was applied. This technique allows for the transformation of positive values, including zeros, while preserving their relative order and reducing the influence of extreme outliers.

#### 3.2.5. Stratified Data Partitioning and Robustness Evaluation

The dataset was divided into three subsets using stratified splitting: training (59.1%, 13,179 records), validation (19.7%, 4394 records), and testing (19.7%, 4394 records). This division maintained the original class distribution (15.6% minority class, 84.4% majority class). To evaluate robustness and generalization capability, this partitioning process was repeated 35 times using different random seeds (0 to 34), generating 35 independent train-validation-test splits. This approach, consistent with recent practices for analyzing variability in model performance [26], allowed us to assess stability across different data configurations while preserving stratification in each split.

#### 3.2.6. Class Imbalance Management

The target variable dejo_declarar exhibits a notable imbalance, with only 15.6% of observations belonging to the minority class. To mitigate the adverse effects of this imbalance during training, particularly in models sensitive to class distribution such as deep neural networks, the synthetic oversampling technique SMOTE (Synthetic Minority Over-sampling Technique) was applied to the training set of each partition, adjusting the ratio to achieve a 50:50 class balance.

This technique generates new synthetic instances of the minority class through interpolations between existing examples, allowing the model to learn more representative patterns of the minority class while balancing the influence of both classes during training. By incorporating SMOTE prior to training, the model’s ability to generalize and correctly discriminate in the presence of imbalanced data is improved, reducing bias toward the majority class. SMOTE was applied exclusively to the training set in each of the 35 partitions. The validation and test sets remained unbalanced to provide realistic performance estimates that reflect the original class distribution.

#### 3.2.7. Variable Scaling

Since deep neural networks are highly sensitive to the scale of input variables, a z-score-based normalization process was applied using StandardScaler. This transformation standardizes each variable to have zero mean and unit standard deviation, which promotes more stable and faster convergence during training, preventing variables with larger numerical scales from dominating the optimization process.

### 3.3. Deep Neural Network Architecture and Training

The predictive model is based on a biologically inspired deep neural network designed to emulate fundamental principles of neuronal information processing. The architecture follows a layered organization analogous to biological neural systems, in which information is progressively transformed through interconnected processing units. Each neuron performs weighted integration of incoming signals followed by nonlinear activation, reflecting synaptic transmission and neuronal firing mechanisms.

With the aim of capturing complex and non-linear patterns in credit behavior, the proposed DNN architecture was implemented in PyTorch 2.5.1 and trained using an automated hyperparameter optimization strategy. To automatically optimize the model’s architecture and hyperparameters of this bioinspired model, the Optuna library was used employing HyperbandPruner as a *pruning* mechanism to early discard less promising combinations.

The search space included the number of hidden layers (1 to 3), the number of units per layer (between 64 and 256, in increments of 64), dropout rates (from 0.2 to 0.5 in increments of 0.1), and L2 regularization coefficients (between $10^{-6}$ and $10^{-2}$), all defined individually per layer. Different activation functions (ReLU and Tanh), optimizers (Adam, RMSprop and SGD) and learning rates (between $10^{-4}$ and $10^{-2}$) were also explored. In total, between 50 and 90 hyperparameter combinations (trials) were evaluated.

Each trial was trained for a maximum of 50 epochs, incorporating an *early stopping* criterion with a patience of 10 epochs without improvement in the validation AUC. In parallel, the Hyperband pruner operated within Optuna to terminate entire trials showing low early performance. Binary cross-entropy was used as the loss function, and the target metric for optimization was the AUC on the validation set. The best model from each trial was stored along with its hyperparameters and the internal state of the training.

This approach enabled the selection of optimal network configurations in terms of predictive capability, while mitigating overfitting through L2 regularization and dropout.

### 3.4. Analysis of Optimal Configurations and Representative Hyperparameter Selection

To identify a representative hyperparameter configuration, the models generated after the tuning process through Hyperband were analyzed, applied independently to each of the 35 stratified partitions of the original dataset. The optimal configurations corresponding to each model were recorded for subsequent comparative analysis.

To group similar configurations, a normalization procedure was applied: continuous numerical values were rounded to five decimal places, and categorical values were standardized as lowercase strings. From these representations, the configuration with the highest frequency of occurrence was identified. This configuration was selected as representative and used to define a model with fixed hyperparameters. The selected configuration corresponded to a biologically inspired deep neural network with three hidden layers of 128, 128, and 64 units, a Tanh activation function, and dropout rates of 0.3, 0.4, and 0.4 per layer. The associated L2 regularization coefficients were $10^{-5}$, $10^{-6}$, and $10^{-5}$, respectively. Training was performed using the Adam optimizer with a learning rate of $2\times 10^{-4}$, minimizing the binary cross-entropy loss function.

This fixed-hyperparameter model was trained in a controlled manner across the 35 available partitions, ensuring model consistency and statistical comparability of the results, as well as robustness to the variability induced by the different partitions of the dataset.

### 3.5. Random Forest Model: Construction, Training, and Evaluation

To establish a rigorous comparison against the biologically inspired deep neural network, a Random Forest model was implemented as a classical, non-bioinspired baseline under the same experimental framework. For each of the 35 stratified partitions of the dataset, the previously generated training, validation, and test subsets were used.

As an initial step, the variable deuda_total was transformed using the adjusted logarithmic function log1p across the three subsets, in order to correct its highly skewed distribution. Subsequently, the predictor variables were scaled using StandardScaler, which was fitted exclusively on the training set and then applied to the validation and test sets, strictly following best practices to prevent data leakage.

To address the class imbalance present in the target variable dejo_declarar, the SMOTE technique was applied exclusively to the training set. This strategy enabled the generation of synthetic instances of the minority class, ensuring a balanced representation during training.

The Random Forest classifier was built using a fixed hyperparameter configuration, selected based on the modal values of the most frequent hyperparameters among the optimal configurations identified in preliminary experiments. This configuration consisted of an ensemble of 400 trees (n_estimators = 400), a maximum depth of 19 levels (max_depth = 19), a minimum of 2 samples required to split an internal node (min_samples_split = 2), a minimum of 1 sample per leaf (min_samples_leaf = 1), and the use of the square root of the number of features as the criterion for predictor selection at each split (max_features = sqrt). This configuration was systematically applied to each of the 35 partitions, enabling a controlled, consistent, and directly comparable evaluation of the model’s performance.

#### Performance Evaluation in Imbalanced Contexts

To evaluate the predictive performance of the developed models, a set of metrics appropriate for scenarios with severe class imbalance was considered. In addition to the area under the ROC curve (AUC), further metrics such as precision, recall, F1-score, Cohen’s Kappa, and Matthews Correlation Coefficient (MCC) were computed. These metrics capture different aspects of model performance, with particular emphasis on the effective detection of the minority class, generalization capability, and adjusted agreement relative to random decisions.

All metrics were calculated on the test set for each of the 35 stratified partitions, for both the deep neural network model and the Random Forest model. This strategy ensured a fair and robust comparison between approaches, accounting for variations due to data partitioning.

### 3.6. Model Interpretability Through Gradient-Based Attributions

Given the limited interpretability and high dimensionality of biologically inspired deep neural network models, gradient-based attribution techniques were incorporated to assess the model’s consistency with respect to the most relevant explanatory variables. In particular, the *Integrated Gradients* (IG) method was applied, which estimates the marginal contribution of each feature to the prediction by integrating the model’s gradient with respect to its inputs along an interpolated path from a neutral reference. This technique enables the generation of both local (per-instance) and global (per-class) explanations, and is applicable to arbitrary architectures as long as they are fully differentiable.

For its implementation, the Captum library from PyTorch was used, specifically designed for the interpretability of neural network models [27]. This tool enabled the extraction of consistent attributions and visualizations that can be integrated into the model evaluation pipeline.

## 4. Results

This section presents the results obtained from the evaluation of the two predictive models considered: a biologically inspired Deep Neural Network (DNN), which constitutes the primary focus of this study, and a Random Forest (RF) classifier used as a baseline model. Both models were trained and evaluated on 35 stratified partitions of the original dataset, generated using different random seeds. The performance metrics were computed on the test set corresponding to each partition, using previously selected fixed hyperparameter configurations.

### 4.1. Evaluation Metrics with Confidence Intervals

Table 2 summarizes the mean values, standard deviations, minimum and maximum values, as well as the 95% confidence intervals for each evaluated metric. The results include the AUC, precision, recall, F1-score, Cohen’s Kappa coefficient, and Matthews Correlation Coefficient.

The bioinspired DNN model achieved an average AUC of 0.9991, with a standard deviation of 0.0003, a minimum value of 0.9984, and a maximum of 0.9996. The 95% confidence interval was ±0.0001. Precision reached a mean value of 0.9977, with a standard deviation of 0.0007 and a range between 0.9960 and 0.9989. Recall showed an average of 0.9956, a standard deviation of 0.0012, a minimum of 0.9935, and a maximum of 0.9984. The F1-score had a mean value of 0.9966, with a standard deviation of 0.0006, a minimum of 0.9950, and a maximum of 0.9980. Both Cohen’s Kappa coefficient and the Matthews Correlation Coefficient reported mean values of 0.9784, standard deviations of 0.0040, and ranges of 0.9682 to 0.9870 in the case of Kappa, and 0.9683 to 0.9870 in the case of MCC. The 95% confidence interval for both metrics was ±0.0014.

In the case of the Random Forest model, the average AUC was 0.9709, with a standard deviation of 0.0026, a minimum of 0.9647, and a maximum of 0.9796. The 95% confidence interval was ±0.0009. Precision reached an average of 0.9714, with a standard deviation of 0.0031, a minimum of 0.9651, and a maximum of 0.9778. Recall showed a mean value of 0.9290, with a standard deviation of 0.0050 and a range from 0.9202 to 0.9412. The F1-score had an average of 0.9497, with a standard deviation of 0.0027, a minimum of 0.9464, and a maximum of 0.9579. Cohen’s Kappa coefficient showed a mean value of 0.7122, a standard deviation of 0.0135, a minimum of 0.6939, and a maximum of 0.7568, with a confidence interval of ±0.0046. The Matthews Correlation Coefficient reported a mean of 0.7182, a standard deviation of 0.0134, a minimum of 0.6990, a maximum of 0.7621, and a confidence interval of ±0.0046.

To assess whether the observed differences between the Deep Neural Network (DNN) and the Random Forest (RF) model were statistically significant, a paired Student’s *t*-test was applied to the AUC, precision, recall, F1-score, Cohen’s Kappa coefficient, and Matthews correlation coefficient. The test was conducted using the same test sets for both models across the 35 stratified partitions.

The results, reported in Table 3, show that the bioinspired DNN significantly outperformed the RF baseline across all evaluated metrics, with *p*-values below 0.01 in every case. These findings indicate that the observed performance differences cannot be attributed to random variation, but instead reflect a consistent and statistically significant improvement of the DNN model over the RF model.

### 4.2. Average ROC Curves

**Figure 1:**
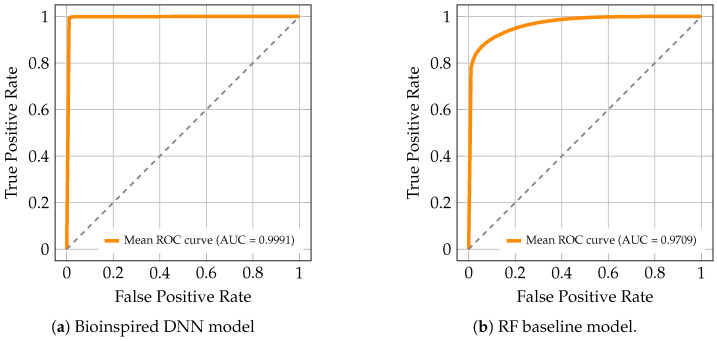
Mean ROC curves of the evaluated models: (**a**) bioinspired DNN and (**b**) RF baseline.

Figure 1a and Figure 1b present the average ROC curves obtained for the Deep Neural Network (DNN) and Random Forest (RF) models, respectively, computed over the 35 stratified partitions of the dataset.

In the case of the bioinspired DNN model (Figure 1a), the ROC curve shows performance close to the upper limit of the metric, with an average area under the curve (AUC) of 0.9991. The trajectory of the curve remains adjacent to the upper-left axis, demonstrating high true positive rates across the entire range of false positive rates.

For the RF model (Figure 1b), the ROC curve shows an average area under the curve (AUC) of 0.9709. The curve consistently lies above the reference diagonal, with a clear separation from randomness across the range of false positive rates, while maintaining high true positive rate values over most of the evaluated interval.

### 4.3. Distribution and Variability of Performance Metrics

**Figure 2:**
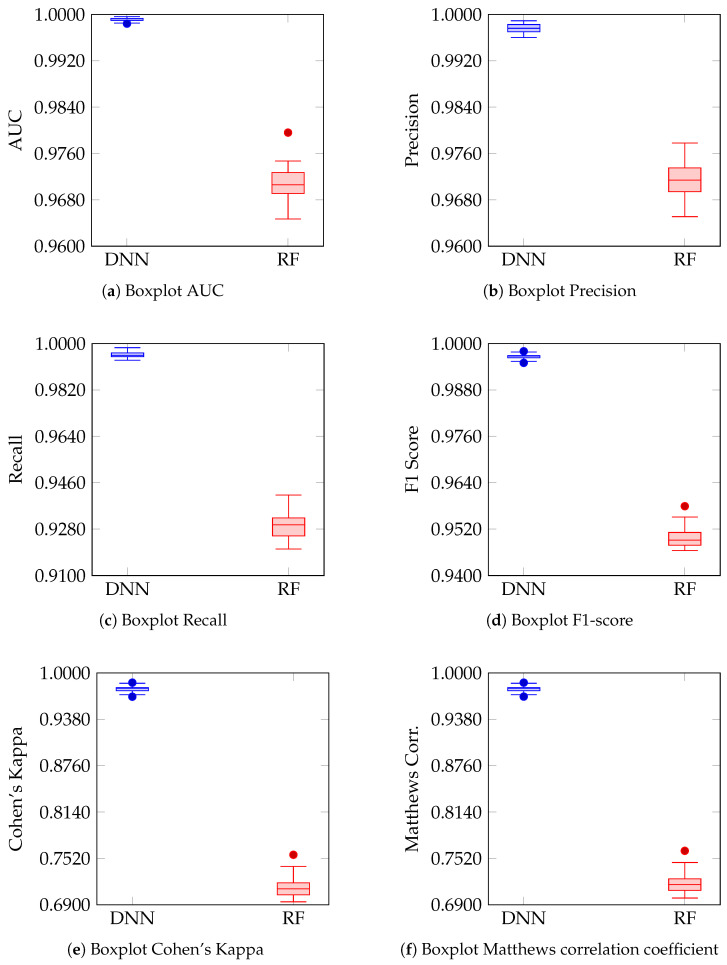
Boxplots of performance metrics computed over 35 stratified dataset partitions for the DNN and RF models: (**a**) AUC; (**b**) Precision; (**c**) Recall; (**d**) F1-score; (**e**) Cohen’s Kappa; (**f**) Matthews correlation coefficient.

Figure 2 show the distribution of the values obtained across the 35 runs for each of the performance metrics of the DNN and RF models. In all plots, the boxplots use distinct colors for each model (blue for DNN and red for RF), enabling a direct and visual comparison of their performance. The consistency in the boxplot design and in the metric scales facilitates the interpretation of differences in precision, recall, F1 Score, Cohen’s Kappa, and the Matthews correlation coefficient between the two approaches.

In Figure 2a, corresponding to the AUC, the boxplots show that the bioinspired DNN model concentrates its values at the upper end of the scale, with minimal variability, while the RF model exhibits a broader dispersion centered in lower ranges. Figure 2b, which represents precision, reflects a similar behavior: the DNN displays a compact and elevated distribution, whereas the RF shows greater spread in its dispersion. In Figure 2c, which illustrates recall, the DNN maintains high values with low variability, in contrast to the RF, which lies at a lower level and with a more extended distribution.

Regarding the F1-score (Figure 2d), the results of the DNN cluster at the upper end of the scale with reduced dispersion, whereas those of the RF display greater spread and lower values. Figure 2e, corresponding to Cohen’s Kappa coefficient, shows that the DNN reaches values distributed toward the upper extreme, while the RF concentrates its results in a noticeably lower range. Finally, in Figure 2f, the boxplots of the Matthews correlation coefficient reproduce the same pattern observed in Kappa: the DNN maintains high values concentrated at the top, whereas the RF lies in a lower range with greater dispersion.

### 4.4. Class-Wise Classification Statistics

Table 4 and Table 5 summarize the statistics of the confusion matrices obtained for the DNN and RF models, respectively, based on the 35 runs performed. The mean, standard deviation, and the minimum and maximum values recorded for each element of the matrix are reported.

In the case of the bioinspired DNN model (Table 4), the true negatives are around an average of 676, with a range from 670 to 681, while the false positives average 9, with a range from 4 to 15. The false negatives reach an average of 16, with values between 6 and 24, and the true positives present an average close to 3692, with a range from 3685 to 3703.

In the RF model (Table 5), the true negatives reach an average of 584, with a range from 560 to 606, while the false positives present an average of 101, with a range between 79 and 125. The false negatives record an average of 264, with values between 218 and 296, whereas the true positives show an average of 3445, with a range from 3413 to 3491.

### 4.5. Feature Attribution Using Integrated Gradients

Feature attributions were calculated using the Integrated Gradients (IG) method applied to the biologically inspired deep neural network model. The analysis was performed on 35 independent partitions of the original dataset, employing 200 samples per partition to estimate the attributions. For each partition, the mean IG attribution per feature was computed for two classes (Class 1 and Class 0), and the mean difference between the classes, as well as the mean absolute difference of the attributions, was obtained.

**Figure 3:**
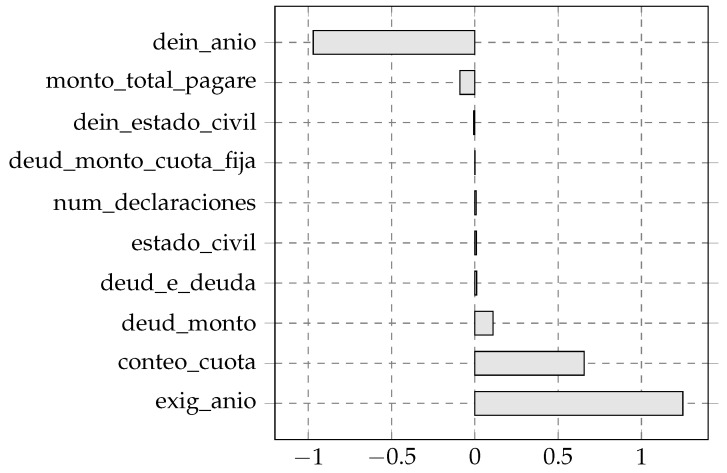
Feature Importance (Integrated Gradients).

Figure 3 displays the ten variables with the highest absolute average importance, according to the mean IG difference between classes. This criterion allowed us to identify the variables with the greatest differential contribution in terms of integrated importance according to IG.

The variable that exhibited the highest mean absolute difference was exig_anio with a value of 1.2487. Next, conteo_cuota and deud_monto showed mean differences of 0.6559 and 0.1096, respectively, being the ones with the largest positive differences. The following variables with positive differences, though of smaller magnitude, were deud_e_deuda (0.0105), estado_civil (0.0086) and num_declaraciones (0.0077).

On the other hand, four variables exhibited negative mean differences: deud_monto_cuota_fija showed a value close to zero (0.0004), followed by dein_estado_civil (−0.0072), monto_total_pagare (−0.0902), and dein_anio, the latter showing the most pronounced negative mean difference (−0.9701).

## 5. Discussion

The comparative evaluation of the models shows that the bioinspired deep neural network (DNN) consistently outperformed the Random Forest (RF) classifier, used as a baseline, in predicting discontinuity in income reporting within the FSCU program. The average AUC of the DNN (0.9991) significantly surpassed that of the RF (0.9709), with parallel improvements in precision, recall, and F1 score. These results suggest that the bioinspired DNN is capable of capturing complex and nonlinear relationships present in the data, aligning with previous findings on deep learning applications in credit risk [28]. Prior research has reported similar advantages of deep networks over traditional models in scenarios characterized by heterogeneous variables and complex relational structures [7,29]. These findings acquire particular importance in the Chilean higher-education context, where institutions such as PUCV must proactively identify irregularities in income reporting to ensure the operational stability of the FSCU loan system.

The exceptionally high performance metrics observed, including a mean AUC of 0.9991 for the DNN, reflect the highly structured nature of administrative student loan records within the FSCU system. Key variables related to debt status, installment dynamics, and declaration timing encode strong compliance signals associated with income-reporting discontinuities. Information leakage was prevented by applying MissForest imputation and SMOTE exclusively to the training data within each of the 35 stratified partitions. Under this evaluation scheme, the risk of overfitting was mitigated through repeated out-of-sample assessment, with no systematic divergence observed between training and test performance. Nevertheless, model performance cannot be assumed invariant under behavioral or regulatory changes, which motivates periodic reassessment in operational settings [9,30].

The robustness of the DNN’s performance was evidenced by the low standard deviation and narrow confidence intervals across all evaluated metrics, indicating stability with respect to variations in the training partitions. The minimal variability observed across the 35 partitions supports the strength of the approach and reduces the likelihood that the results are attributable to favorable splits [31]. This robust behavior aligns with studies emphasizing the importance of multiseed evaluations to ensure reproducibility in classification systems applied to financing and risk [31,32]. The consistency achieved is particularly relevant in financial contexts, where the replicability and reliability of predictions directly affect risk management decision-making [33,34].

The superiority of the bioinspired DNN over the RF baseline was further supported by paired Student’s *t*-tests conducted across the 35 stratified partitions. Statistically significant differences were observed for all evaluated metrics, including AUC, precision, recall, F1-score, Cohen’s Kappa, and Matthews Correlation Coefficient (*p* < 0.001 in all cases). These results confirm that the observed performance gains are systematic and not attributable to random variation across data partitions.

The class-wise analysis highlighted the operational relevance of the bioinspired DNN [31]. The reduction in false negatives (16.49) and false positives (8.63) compared to RF (FN = 263.51; FP = 101.40) significantly decreases the risk of incorrect credit decisions, enabling the calibration of decision thresholds according to institutional policies [35]. This finding underscores the importance of complementing global metrics such as AUC with class-specific error indicators to optimize credit risk management and minimize negative impacts on beneficiaries [33,34]. In the specific case of Chilean universities operating under the FSCU regime, the reduction in both types of misclassification is directly linked to improved financial planning and more efficient allocation of institutional resources devoted to credit risk management.

The preprocessing pipeline implemented contributed decisively to the performance observed. Imputation with MissForest, variable transformations, and class balancing using SMOTE enabled the DNN to learn representative patterns without biases arising from missing data or class imbalance [17,20]. This systematic approach is consistent with good practices in financial risk modeling and reinforces the need for careful data treatment prior to training complex models [32,33].

In terms of computational efficiency, all experiments were conducted on a system equipped with a 13th-generation Intel Core i7-13620H processor (10 cores, 16 threads) and 16 GB of RAM. The average training time of the deep neural network was approximately 1.47 min per partition, whereas the Random Forest model required around 0.03 min. This difference reflects the higher computational complexity associated with optimizing the weights of the neural network compared to the construction of trees in the Random Forest. Nevertheless, given that model training is performed offline and inference requires negligible computational resources, the observed training times are compatible with periodic institutional risk assessment cycles rather than real-time decision systems.

The interpretability analysis using Integrated Gradients indicated that the most influential variables were exig_anio, conteo_cuota, deud_monto, and dein_anio, reflecting the model’s reliance on financial and compliance-related indicators aligned with FSCU monitoring policies. Variables with positive contributions increased the probability of predicting income reporting discontinuities, whereas negative contributions favored compliance predictions [36]. Demographic variables exhibited minimal influence, suggesting a low risk of indirect bias and supporting fairer deployment in student credit monitoring systems. These attributions describe relative feature importance without implying causal relationships [37,38].

The evaluation using complementary agreement metrics, such as Cohen’s Kappa (0.9784) and the Matthews Correlation Coefficient (0.9784), supported the high consistency between predictions and true labels, providing a more comprehensive perspective in the presence of class imbalance [22,39]. Incorporating multiple metrics enhances the transparency of the analysis and represents a recommended practice in credit risk studies involving imbalanced populations [32,34,40].

Despite the robustness of the experimental design based on repeated stratified partitions, the analysis relies on a single institutional dataset from the Chilean FSCU program, limiting external and temporal generalizability. Such constraints are common in administrative credit risk studies subject to regulatory and data-access restrictions. The contribution is therefore methodological rather than institutional. The proposed pipeline, including leakage-controlled preprocessing, imbalance handling restricted to the training data, and multiseed validation, is transferable to other student loan systems. External validation across institutions and cohorts is required to assess robustness under distributional and policy-induced shifts [30,41].

Finally, the findings suggest that the bioinspired DNN constitutes a promising approach for credit risk prediction in student financing programs. It is recommended to prioritize external validation, sensitivity analyses on preprocessing strategies, threshold calibration, and periodic audits of fairness and performance. The methodology presented—including the pipeline, the evaluation over 35 partitions, and the interpretability analysis—provides a replicable framework for future applications in institutional contexts.

## 6. Conclusions

This work addressed the prediction of discontinuity in income reporting within the Chilean FSCU program, a central component of the student-financing system administered directly by universities such as the Pontificia Universidad Católica de Valparaíso, focusing on a bioinspired deep neural network (DNN) as the primary predictive model. Although the literature has incorporated models capable of capturing complex relationships, a gap remains in methodologically robust evaluations that integrate bioinspired deep learning approaches, advanced imbalance handling, nonlinear imputation, and repeated validation across multiple partitions. To address this gap, a Deep Neural Network (DNN) model was developed and evaluated, and its performance was compared with that of a Random Forest (RF) classifier using a preprocessing pipeline that included MissForest imputation, SMOTE balancing, and consistent feature transformations. By being grounded in the administrative datasets of PUCV, the proposed methodology reflects the operational conditions faced by Chilean institutions responsible for managing FSCU portfolios, thereby strengthening its practical relevance.

The results showed that the bioinspired DNN consistently outperformed the RF across all evaluated metrics. The bioinspired DNN achieved an average AUC of 0.9991, compared to 0.9709 for the RF baseline, with similar improvements observed in precision, recall, F1-score, and agreement metrics such as Cohen’s Kappa and the Matthews Correlation Coefficient. The bioinspired DNN also exhibited substantially lower variability and clear reductions in both false positives and false negatives, indicating greater stability and reliability for supporting credit decision-making. Interpretability analysis of the bioinspired DNN using Integrated Gradients revealed that variables related to debt structure and administrative compliance contributed most strongly to the model’s predictions, while demographic variables showed minimal impact, thereby reducing the risk of biases associated with personal characteristics.

Among the study’s limitations, the absence of external or temporal validation stands out, restricting the generalization of results to other contexts or periods. Additionally, although SMOTE and MissForest improved model performance, the sensitivity to alternative preprocessing strategies was not explored in depth.

For future work, we recommend validating the model on external datasets and across different time periods, exploring alternative imputation and balancing techniques, adjusting and calibrating decision thresholds according to institutional policies, and conducting periodic fairness and performance audits to ensure transparency and replicability. In the Chilean context, such measures are essential to support evidence-based decision-making in universities that rely on FSCU repayment flows as part of their financial structure. The methodology presented, centered on a bioinspired DNN, constitutes a robust and replicable framework for credit-risk prediction in student support programs and other institutional financial contexts.

## Figures and Tables

**Table 1:** Variables with Significant Missing Values.

Variable	Null (n)	Data Type	% of Nulls
sexo	7414	object	33.24%
fecha_nacimiento	7446	datetime64[ns]	33.39%
deud_monto_cuota_fija	6009	float64	26.94%
deuda_total	336	float64	1.51%

**Table 2:** Evaluation Metrics with 95% Confidence Intervals.

Model	Metrics	Mean	Desv. Std	Min	Max	IC 95%
DNN	AUC	0.9991	0.0003	0.9984	0.9996	0.9991 ± 0.0001
	Precision	0.9977	0.0007	0.9960	0.9989	0.9977 ± 0.0003
	Recall	0.9956	0.0012	0.9935	0.9984	0.9956 ± 0.0004
	F1 Score	0.9966	0.0006	0.9950	0.9980	0.9966 ± 0.0002
	Cohen’s Kappa	0.9784	0.0040	0.9682	0.9870	0.9784 ± 0.0014
	Matthews Corr.	0.9784	0.0040	0.9683	0.9870	0.9784 ± 0.0014
RF	AUC	0.9709	0.0026	0.9647	0.9796	0.9709 ± 0.0009
	Precision	0.9714	0.0031	0.9651	0.9778	0.9714 ± 0.0011
	Recall	0.9290	0.0050	0.9202	0.9412	0.9290 ± 0.0017
	F1 Score	0.9497	0.0027	0.9464	0.9579	0.9497 ± 0.0009
	Cohen’s Kappa	0.7122	0.0135	0.6939	0.7568	0.7122 ± 0.0046
	Matthews Corr.	0.7182	0.0134	0.6990	0.7621	0.7182 ± 0.0046

**Table 3:** Results of the paired Student’s *t*-test between the bioinspired DNN and RF baseline models.

Metric	*t*-Value	*p*-Value
AUC	62.71	0.00
Precision	47.56	0.00
Recall	83.48	0.00
F1 Score	107.66	0.00
Cohen’s Kappa	118.66	0.00
Matthews Corr.	116.56	0.00

**Table 4:** Confusion matrix of the bioinspired DNN model.

Element	Mean	Desv. Est.	Minimum	Maximum
True Negative (TN)	676.37	2.74	670	681
False Positive (FP)	8.63	2.74	4	15
False Negative (FN)	16.49	4.64	6	24
True Positive (TP)	3692.49	4.63	3685	3703

**Table 5:** Confusion matrix of the RF baseline model.

Element	Mean	Desv. Est.	Minimum	Maximum
True Negative (TN)	583.60	11.58	560	606
False Positive (FP)	101.40	11.58	79	125
False Negative (FN)	263.51	18.46	218	296
True Positive (TP)	3445.46	18.44	3413	3491

## Data Availability

The raw data supporting the conclusions of this article will be made available by the authors on request.
